# The Influence of Serotonergic Signaling on Quality of Life, Depression, Insomnia, and Hypoxia in Obstructive Sleep Apnea Patients: Cross-Sectional Study

**DOI:** 10.3390/jcm14020445

**Published:** 2025-01-12

**Authors:** Marta Ditmer, Agata Gabryelska, Szymon Turkiewicz, Adrian Gajewski, Piotr Białasiewicz, Maciej Chałubiński, Dominik Strzelecki, Alicja Witkowska, Marcin Sochal

**Affiliations:** 1 Department of Sleep Medicine and Metabolic Disorder, Medical University of Lodz, 6/8 Mazowiecka, 92-215 Lodz, Poland; 2 Department of Immunology and Allergy, Medical University of Lodz, 251 Pomorska, 92-213 Lodz, Poland; 3 Department of Affective and Psychotic Disorders, Medical University of Lodz, 251 Pomorska, 92-213 Lodz, Poland

**Keywords:** OSA, sleep, serotonin, 5-HT, SERT

## Abstract

**Background/Objectives**: Serotonin and the serotonin transporter (SERT) may have a multifaceted, but not fully understood, role in obstructive sleep apnea (OSA) and its impact on mental health in this group of patients. This study aimed to investigate changes in serotonin and the serotonin transporter (SERT) and their association with depressive and insomnia symptoms. **Methods**: This study included 76 participants (OSA group: *n* = 36, control group (CG): *n* = 40) who underwent polysomnography, while venous blood samples (evening and morning) were analyzed for serotonin and the SERT using ELISA. SERT mRNA expression in peripheral leukocytes was measured via quantitative reverse-transcription polymerase chain reaction (qRT-PCR). Participants were evaluated for depression, insomnia, and quality of life (QoL). **Results**: This study found no significant differences in SERT mRNA or serotonin between the OSA group and CG. In the CG, individuals without mood disorders had higher baseline SERT levels and evening/morning SERT ratios than those with depression. Among the OSA participants, those with good QoL had elevated serotonin levels in the evening (*p* = 0.028) and morning (*p* = 0.043) compared to those with poor QoL. Baseline SERT protein levels were higher in the CG than in the OSA group for insomnia, while SERT mRNA expression was higher in the OSA group. Linear regression models showed 13.3% and 13.1% for non-rapid eye movement sleep (NREM) apnea/hypopnea index (AHI) and AHI variability, respectively, which was accounted for by the morning SERT level, while 30.8% of the arousal index variability was explained by the morning serotonin level. **Conclusions**: Serotonergic signaling may influence quality of life, depression, and insomnia in OSA, as well as the severity of the disease itself. Stratifying patients by clinical and laboratory phenotypes could enable more personalized treatment.

## 1. Introduction

Obstructive sleep apnea (OSA) is a heterogeneous disorder characterized by repeated partial to complete upper airway blockages during sleep [1]. It instigates systemic effects, such as arousals, sleep fragmentation, and desaturations, which further lead to intermittent hypoxia and hypercapnia. Diagnosis of OSA is established through polysomnography (PSG) examination, with severity assessed using the apnea/hypopnea index (AHI) [2]. The standard treatment is continuous positive airway pressure (CPAP) applied to the upper airways [3]. The common risk factors for OSA include obesity, male sex, age from 40 to 70 years, and anatomical abnormalities [4]. However, the pathogenesis of OSA is multifactorial, extending beyond these factors to include genetic predispositions and dysfunctions in ventilatory control and neuromuscular tone, which are regulated by neurohormonal agents such as serotonin (5-hydroxytryptamine, serotonin) [5]. In the central nervous system, this neurotransmitter is primarily produced in the raphe nuclei of the brainstem [6]. Serotonergic neurons project to the hypoglossal nucleus in the brainstem, providing excitatory input to the upper airway motor neurons [7]. This allows for the maintenance of the patent airways by dilator muscles, the largest of them being the genioglossal muscle [6,7]. The activity of serotonergic motor neurons that innervate the brainstem is decreased during sleep, particularly during REM, which contributes to upper airway obstruction [7]. In adult rats, during both REM and NREM sleep, within the hypoglossal nucleus, the microperfusion of serotonin does not allow for complete muscle relaxation and airway collapse [7]. In the periphery, 5-HT2A, 5-HT2C, and 5-HT3 receptors exert an inhibitory effect on respiration via the nodose ganglion.

The relationship between OSA and serotonin appears to be mutual; intermittent hypoxia has been shown to promote serotonergic innervation of the hypoglossal nuclei, suggesting the existence of a feedback loop [8].

Beyond its role in modulating respiratory stability, serotonin has been implicated in the broader pathophysiology of OSA, linking upper airway dysfunction to systemic effects [9]. Interestingly, growing evidence highlights the associations between OSA and psychiatric comorbidities, such as anxiety and depression, underscoring the potential interplay between serotonin modulation and OSA pathophysiology [10,11,12]. As a heterogenous disorder, OSA presents a spectrum of phenotypes that differ in clinical presentation and underlying mechanisms [13]. The insomnia-predominant phenotype is characterized by challenges in initiating or maintaining sleep and is associated with psychiatric conditions such as depression and anxiety. This phenotype underscores the connection between disrupted sleep and mood regulation, which is particularly relevant for studies examining the relationship between OSA and psychiatric disorders [14].

Serotonin is a monoamine neurotransmitter of the central nervous system (CNS) synthesized from L-tryptophan by tryptophan hydroxylase 1 (TPH1) and further transformed into melatonin (*N*-acetyl-5-methoxytryptamine) [15]. It bears significant implications for maintaining cognition, mood, and sleep [16]. Notably, serotonin particularly stimulates the hypoglossal nerve, which is essential for maintaining oropharyngeal patency by preserving genioglossus muscle tone [17]. Additionally, serotonin contributes to regulating CO_2_ and pH homeostasis through its chemosensitive properties [18].

The serotonin transporter (SERT) mediates the reabsorption and further interaction with the concentration of serotonin in the CNS via multiple receptors [19]. Polymorphisms of SERT like the 5-hydroxytryptamine transporter variable-number tandem repeat (5-HTTVNTR) and 5-hydroxytryptamine transporter gene-linked polymorphic region (5-HTTLPR) have been shown to predispose to the OSA development [20,21]. These genetic variations are also implicated in the pathogenesis of depression [22]. This shared genetic basis only emphasizes the dependencies between depression and anxiety in individuals with OSA. Furthermore, the SERT is the pharmacological target of selective serotonin reuptake inhibitors (SSRIs), which are widely used in the treatment of depression and anxiety [23]. Notably, SSRIs have also been explored as potential adjunctive therapies in the management of OSA, highlighting the intricate relationship between serotonergic pathways, mood disorders, and sleep apnea [21,24]. However, the outcomes of these studies have been inconsistent, likely due to several factors, including small sample sizes, insufficient duration of observation, and the heterogeneity of OSA. Since the SERT, in essence, is one of two possible ways of terminating serotonin’s action in a synapse (the other one being degradation by monoamine oxidase A), it is reasonable to suspect that alongside its ligand it may have an important role in the pathophysiology of OSA [6].

Despite advancements in evaluating serotonin’s influence on respiratory and neural functions [25], the interplay between serotonin modulation and the pathophysiology of OSA remains insufficiently understood. Research on sleep-disordered breathing (SDB), encompassing conditions such as OSA, sleep-related hypoventilation, hypoxemia, and snoring has revealed an association between SDB and reduced serum serotonin levels. Notably, serum serotonin levels have been found to correlate negatively with the oxygen desaturation index, as well as central and obstructive apnea scores [26]. Furthermore, the use of CPAP therapy for three months has been shown to improve sleep patterns and significantly elevate serum serotonin levels [27]. Moreover, intermittent hypoxia, the primary pathological factor in OSA, has been reported to impair peripheral serotonin uptake and degradation in animal models [28]. However, existing studies have primarily focused on generalized OSA models, with limited attention to specific phenotypes such as the insomnia-predominant subtype and its correlation with the serotonergic pathway. The role of serotonin in depression and insomnia on OSA remains elusive.

Thus, this study aimed to investigate the associations between serotonin and SERT levels in the context of insomnia and depression among OSA patients. Additionally, the study sought to explore correlations between these parameters and OSA severity, as well as to compare OSA patients with non-OSA participants to identify potential differences and underlying mechanisms.

## 2. Materials and Methods

A total of 76 participants were recruited at the Department of Sleep and Metabolic Disorders, Medical University of Lodz, Poland. The sample was collected over a period of 4 years; no sample size calculations were conducted prior to the study.

Inclusion criteria were specified as the following: written informed consent to participate in the study, age between 18 and 75 years with a body mass index (BMI) ranging from 20 to 45 kg/m^2^. The exclusion criteria included inflammatory or chronic respiratory diseases, recent infections within one month before blood collection, a history of cancer, neurological disorders, diagnosed psychiatric conditions, current psychiatric treatment (medications affecting histamine H1 receptor, noradrenaline, dopamine, serotonin, and GABA gamma-aminobutyric acid (GABA) signaling), and insomnia requiring active treatment. All individuals who met the inclusion criteria underwent diagnostic PSG to assess sleep-related parameters. Based on the AHI calculated from the PSG results, participants were divided into two groups: those diagnosed with severe OSA having an AHI greater than 30 and a control group with an AHI less than 5.

Upon admittance to the department at 21:00 (±0.5 h), participants underwent a physical examination that included measurements of height, weight, heart rate, and blood pressure. Following the examination, overnight PSG was conducted. The PSG recordings incorporated multiple channels for comprehensive monitoring: electroencephalography (C4\A1, C3\A2); electromyography of the chin and anterior tibialis muscles; electrooculography; thermistor gauges to measure oronasal airflow; snoring sound recordings; body position sensors to monitor sleep posture; piezoelectric gauges to detect respiratory movements of the chest and abdomen; a unipolar electrocardiogram or heart activity; and measurements of hemoglobin oxygen saturation (SpO_2_) using the Alice 6 system by Phillips-Respironics.

Sleep stages were analyzed in 30 s epochs according to the guidelines established by the American Academy of Sleep Medicine [29]. An apnea event was characterized by a reduction in airflow to less than 10% of the baseline for a minimum of 10 s. Hypopnea was defined as at least a 30% decrease in airflow lasting at least 10 s, accompanied by an SpO_2_ reduction exceeding 3% or arousal.

Peripheral venous blood for the biochemical examination was collected in the evening before PSG and in the morning after PSG in collection tubes with a clot activator and EDTA. Blood samples with a clot activator were centrifuged immediately following blood draws at 4 °C. Obtained serum and whole blood in EDTA tubes were stored at −80 °C.

Participants underwent assessments of depressive symptoms using chosen questionnaires and scales in the morning after the PSG.

The Beck Depression Inventory (BDI) is a 21-item questionnaire designed to evaluate the severity of depressive symptoms. Each item offers at least four response options, varying in intensity and scored on a scale from 0 to 3. A score of 11 or higher is considered the threshold for mild depression, but the results are not intended to provide a definitive diagnosis of depression [30]. In the present study, the cut-off point for significant depressive symptoms was more than 13 points.

The Insomnia Severity Index (ISI) is a 7-item questionnaire designed to evaluate the severity of insomnia symptoms and their impact on daily life. Each item is rated on a 5-point Likert scale, resulting in a total score ranging from 0 to 28. Scores are categorized to reflect no clinically significant insomnia, subthreshold insomnia, moderate insomnia, or severe insomnia. The ISI is not intended to diagnose insomnia but is widely used to assess symptom severity and monitor treatment outcomes [31]. The cut-off point for significant insomnia symptoms was placed at >14 points.

The SF-36 Health Survey (SF-36) is a 36-item questionnaire designed to measure health-related quality of life across eight domains, including physical functioning, bodily pain, general health perceptions, vitality, and mental health. Each domain is scored on a scale from 0 to 100, with higher scores indicating better health or functioning. The SF-36 does not provide a diagnosis but offers a comprehensive assessment of an individual’s perceived physical and mental well-being [32]. The cut-off point differentiating between good and poor life quality was placed at a score either equal/higher or lower than 64 points, respectively.

RNA was isolated from peripheral blood leukocytes using the TRIzol reagent (Invitrogen, Waltham, MA, USA). The RNA Integrity Number (RIN) and RNA concentration were measured using a Nanodrop Colibri Micro-volume Spectrometer (Titertek Berthold, Bad Wildbad, Germany). The isolated RNA was then reverse transcribed into complementary DNA (cDNA) using the SuperScript IV First-Strand Synthesis System (Thermo Fisher Scientific Inc., San Jose, CA, USA) following the manufacturer’s protocol. This process included three steps, with annealing performed at 60 °C for 60 s. Gene expression levels were quantified using quantitative real-time polymerase chain reaction (qRT-PCR). The reaction mix contained nuclease-free water, TaqMan Universal Master Mix, cDNA, and gene-specific TaqMan probes (target gene: SERT; reference gene: β-Actin). Each sample and reference gene underwent three replicates. The cycle threshold (Ct) value was determined for every sample, followed by the calculation of ∆Ct, which was subsequently analyzed using the Livak (2^−∆∆Ct^) method to determine mRNA expression levels [33].

The serum levels of serotonin and SERT were assayed at the Department of Sleep Medicine and Metabolic Disorders, Medical University of Lodz, Poland, using the enzyme-linked immunosorbent assay (ELISA) method. Serotonin was determined with the Dermeditec test (Kiel, Germany) and SERT with the Elab test (Nanjing, China). Absorbance was assessed using the 450 nm spectrum of the 800TS Absorbance Reader (Agilent Technologies, Santa Clara, CA, USA). Additionally, evening/morning ratios of serum serotonin, SERT protein, and SERT mRNA were calculated.

The study was accepted by the Ethical Committee of the Medical University of Lodz, Poland, with the following referencing number: (RNN/432/18/KE). Moreover, it was designed in line with the declaration of Helsinki, and all procedures were performed in accordance with relevant guidelines and regulations.

Statistical analyses were performed using Statistica 13.1 PL (StatSoft, Tulsa, OK, USA). A *p*-value of less than 0.05 was considered statistically significant. The Shapiro–Wilk test was used to assess the normality of data distributions. Variables that were normally distributed (evening SERT protein level in CG with either poor or good life quality) were compared using dependent *t*-tests for paired samples and independent *t*-tests, while non-normally distributed variables (other measured parameters in created subsets of participants) were analyzed using the Wilcoxon and Mann–Whitney U tests. Nominal variables (i.e., sex) were evaluated with chi-square tests. Spearman’s rank correlation coefficient was employed to examine relationships between continuous variables (i.e., age, BMI, selected OSA severity parameters, questionnaire scores, expression of genes, and level of proteins). Linear regression using a forward stepwise model was conducted to determine the impact of dependent data, including SERT, serotonin, and SERT mRNA, on selected OSA parameters and questionnaires.

## 3. Results

Participants were divided into two study groups: the severe obstructive sleep apnea (OSA) group (*n* = 36) and the control group (CG; *n* = 40) groups. Demographic data, polysomnography results, questionnaire scores, and SERT and serotonin levels for both groups are summarized in Table 1. The two groups were comparable in all demographic variables except BMI, which was higher in the OSA group, and the percentage of women, which was higher in the CG (*p* < 0.001 and *p* = 0.025, respectively; Table 1).

In the OSA group, 22 participants (61.1%) were diagnosed with arterial hypertension, 2 (5.6%) with asthma, 1 (2.8%) with chronic obstructive pulmonary disease, 30 (30.6%) with type 2 diabetes mellitus, and 1 (2.8%) with gastroesophageal reflux disease.

Regarding sleep parameters, the OSA group exhibited increased AHI, oxygen desaturation index (ODI), and arousal index compared to the CG (all *p* < 0.001; Table 1).

No significant differences in serotonin levels, SERT protein levels, SERT mRNA gene expression, or their respective evening/morning ratios were observed between the OSA and CG at any time point (Table 1).

In all groups, except for individuals with reduced quality of life in the OSA group and those with pronounced insomnia symptoms in the CG, morning SERT levels were higher than evening levels (all *p* < 0.05; Table 2).

In the OSA group, participants with a good quality of life (QoL) had significantly higher serotonin levels in both the evening (*p* = 0.028; Table 2) and the morning (*p* = 0.043; Table 2) compared to those with poor life quality. No such differences were found for SERT levels or within the CG. Baseline serum serotonin levels positively correlated with the SF-36 questionnaire score, whereas the A/B SERT ratio correlated negatively with this parameter (R = 0.573, *p* = 0.001 and R = −0.368, *p* = 0.038, respectively).

In the OSA group, depressive symptoms were not associated with changes in any of the studied variables (Table 2). In contrast, within the CG, participants without mood disturbances showed elevated baseline SERT protein levels compared to those with pronounced depressive symptoms (*p* = 0.044; Table 2). Additionally, the evening/morning SERT ratio was significantly higher in this subgroup (*p* = 0.005; Table 2) compared to their CG counterparts with depressive symptoms.

In the insomnia group, baseline SERT protein levels were significantly lower in the OSA group compared to the CG (*p* = 0.045; Table 2). However, baseline SERT mRNA expression levels were higher in the OSA group vs. CG (*p* = 0.032; Table 2). Within the CG group, subjects with good sleep quality exhibited higher baseline SERT mRNA gene expression (*p* = 0.037; Table 2) than those who experienced pronounced insomnia symptoms. No differences were detected in other measured parameters, such as serotonin levels or morning SERT levels.

In the OSA group, SERT protein levels at the two time points were positively correlated with the AHI and NREM AHI. Similar correlations were observed for the arousal index, as well as morning SERT and serotonin levels (Supplementary Table S1).

Linear regression models using a progressive stepwise method were applied to the OSA group. These analyses revealed that the variability in the NREM AHI and AHI were explained by morning serum SERT, with 13.3% and 13.1%, respectively. Furthermore, the 30.8% of the arousal index variability was explained by morning serum serotonin levels (Table 3).

## 4. Discussion

In the existing body of literature, studies investigating OSA phenotypes distinguished by various biochemical and psychological parameters are scarce. The subject of serotonergic signaling and its impact on the overall clinical picture in this group of patients is even less explored. The interplay between this mode of neurotransmission and the pathophysiology of OSA appears to be a complex topic that is yet to be fully understood.

In the present study, no differences were found between the OSA and control groups regarding any of the studied elements of serotonergic signaling; to the best of our knowledge, at the time of the publication no other similar studies were conducted to allow for comparisons. However, distinctions between the groups emerged after the participants were further categorized based on their questionnaire results.

In terms of QoL, it was observed that among individuals with OSA, those reporting higher QoL had elevated serotonin levels at both time points compared to those with lower QoL despite a similar serotonin ratio between the two groups. Moreover, baseline serotonin levels correlated positively with the SF-36 score, whereas the A/B SERT ratio correlated negatively with this parameter. Several authors have already reported a relationship between blood serotonin and well-being; however, none of them used the questionnaire applied in the present study or involved OSA participants. In a study by Tsai et al., SERT availability in the midbrain, as measured in vivo using single-photon emission computed tomography, correlated positively with well-being, assessed by a questionnaire curated by the World Health Organization, but only in males [34].

On the other hand, it was noted that the CG with depressive symptoms presented lower baseline SERT levels, as well as higher SERT morning/evening ratio, than their CG counterparts with better mood. Such a pattern was not observed within the OSA group. There are no other studies involving the assessment of SERT protein levels in OSA patients; however, such outcomes in the CG align with those obtained by evaluating the role of the SERT in the pathophysiology of depression. Several authors, using imipramine as a SERT-binding agent, demonstrated that the level of this protein seems to be decreased in the platelets of individuals suffering from depression [35,36,37]. Studies that focus on psychiatric aspects of serotonergic signaling prefer to use in vivo imaging techniques, such as SPECT or post-mortem analysis. This allows for the assessment of changes within different brain regions, which gives a more nuanced insight into the pathophysiology of depression. Indeed, Kambeitz et al., in their meta-analysis involving both in vivo and post-mortem studies, concluded that serotonin binding indeed appears to be reduced in the limbic system [37]. This allows inferring that OSA could disturb serotonergic signaling in the brain, affecting the pathophysiology of depression.

Concerning sleep problems, it was noted that among individuals with insomnia, baseline SERT protein levels were significantly higher in the CG group compared to the OSA group; however, SERT mRNA expression was more intensive in the OSA group. Within the CG group, subjects who reported good sleep exhibited higher baseline SERT mRNA gene expression than those who experienced pronounced insomnia symptoms. In contrast to our previous study on inflammatory bowel disease patients, SERT protein did not positively correlate with insomnia severity [38]. Such results are difficult to explain in the context of the current literature, as there are no similar studies to draw comparisons. Nevertheless, insomnia is a problem commonly encountered in OSA patients, which seems to bear an association with serotonergic signaling [39]. Deuschle et al. revealed that primary insomnia was associated with a certain allele of the serotonin transporter gene [40]. What is also interesting is that the relationship between the severity of OSA and insomnia is not linear, with studies showing varying results [41]. Perhaps excessive daytime sleepiness, a hallmark of OSA, might skew questionnaire results. Additionally, differences between gene expression and protein level suggest that the process of translation might be disrupted by a range of factors related to hypoxia, sleep, and mental health.

There could be several reasons explaining distinctive patterns of serotonin, SERT protein, and SERT mRNA synthesis across OSA phenotypes, as stratified by questionnaires.

First, changes in the evening/morning SERT ratio in CG with depression, which were not present in the OSA group, as well as a negative correlation between this parameter and the SF-36 questionnaire score, allow suspecting that circadian rhythms might be an important factor in the interplay between OSA and mental health. Indeed, our previous studies seem to confirm that OSA subjects have a dysregulated circadian clock, which might be linked to depression [42,43].

Second, OSA may influence neurotrophin production, potentially disrupting serotonergic signaling and contributing to mental health issues [44]. Among neurotrophins, brain-derived neurotrophic factor (BDNF) is most closely linked to OSA, with altered BDNF signaling associated with complications such as cardiovascular diseases and psychiatric disorders, including sleep disturbances, cognitive impairment, and depression [44]. Flores et al., in their study, found that individuals with OSA had elevated levels of this neurotrophic factor compared to the control group, which could suggest that intensification of its production is a compensatory mechanism, perhaps delaying dementia in those patients [45]. Serotonergic pathways regulate BDNF’s expression and release in the central nervous system, while the discussed neurotrophin in turn supports serotonergic neuron development, maintenance, and plasticity, affecting serotonin synthesis and availability [46]. Moessner et al. demonstrated that BDNF can modulate the SERT-dependent serotonin uptake in lymphoblasts, preferentially in cells with long/long genotypes of SERT [47]. Imbalances in serotonin can impact BDNF levels, and both contribute to synaptic modulation and neuroplasticity, critical in mood disorders like depression [46]. Aguilera et al., in their study, showed that the influence of early-life adversities on depression in adults might be moderated by BDNF Val66Met and SERT gene polymorphisms [48].

Third, intermittent hypoxia—a hallmark of OSA—could influence elements of the serotonergic signaling pathway. In the present study, linear regression analysis identified morning SERT protein levels as the only reliable predictors for AHI and NREM AHI in the OSA group, while the arousal index was best predicted by morning serotonin levels. Other authors also demonstrated that hypoxia might not only inhibit serotonin synthesis but also increase its uptake [25,28,49,50,51]. In Gulf toadfish, exposure to mild hypoxia (oxygen saturation 50%) resulted in a decrease in plasma serotonin by 40% and an increase in serotonin uptake; both changes persisted throughout 24 h [28]. In patients with epilepsy, those who did not experience ictal central apnea had higher levels of serum serotonin than those who did [52]. On the other hand, in patients with partial seizures, intake of selective serotonin reuptake inhibitors decreased the likelihood of ictal oxygen desaturation [53]. It is important to note that during intense seizure activity, the permeability of the blood–brain barrier increases, possibly allowing peripheral serotonin to enter the central nervous system [52].

To date, only one other study has explored the serotonin pathway in OSA subjects. Wieckiewicz et al. reported negative correlations between serotonin and ODI, as well as the number of apneic episodes (central or obstructive) [26]. However, their study did not assess other parameters, such as SERT protein levels, mRNA expression, or the arousal index [26]. Differences between these findings and the present study may be partially attributed to the greater sample size (*n* = 105), a higher proportion of women (76.2%), and the absence of a control group [26]. Serotonergic signaling has been shown to moderate respiration both in the central nervous system and on the periphery. In animal models, exogenous intraperitoneal administration of serotonin resulted in an increased number of central apneas during REM sleep [54]. On the other hand, injection into the external carotid artery transiently elevated respiratory rate while decreasing tidal volume [55]. It might also exert either excitatory or inhibitory effects on medullary respiratory neurons. In rats, administration of mirtazapine, a tetracyclic antidepressant that also works as a serotonin receptor antagonist, reduced the number of central apneic events during REM and NREM sleep [56]. Several authors observed similar effects in humans; however, weight gain, one of the most common side effects of this medication, negates any potential benefits [57]. Aung et al., in their recent review on the subject of serotonergic signaling in sleep-disordered breathing, concluded that the lack of specificity of many 5-HT receptor-targeting drugs raises concerns about their effects on other receptor subtypes and neuronal populations [6]. Additionally, the heterogeneity in OSA severity and patient response complicates predicting treatment outcomes [6]. Nevertheless, searching for adjuvant pharmacotherapies for OSA seems validated.

Both OSA and chronic obstructive pulmonary disease (COPD) are prevalent lung diseases with major systemic complications [58]. A subset of patients might suffer from both, which is referred to as an overlap symptom [58]. Authors suggest that the prevalence of OSA might be higher in COPD patients; however, study results vary [58]. COPD might exacerbate OSA, enhancing ventilation–perfusion mismatch and hypoventilation, which can lead to hypoxia and hypercapnia [58]. Lau et al. and later Pirina et al. demonstrated that patients with COPD have higher circulating serotonin levels than controls [59,60]. Moreover, Lau et al. observed a negative correlation between serotonin levels, FEV1, and FVC, suggesting the role of serotonergic signaling in the severity of obstruction [60]. Such results are in stark contrast with those discussed above. It is possible that in COPD platelets are persistently activated, releasing serotonin into the bloodstream [60]. Indeed, smoking, the most common cause of COPD was found to be associated with platelet activation [60].

Both COPD and OSA might cause pulmonary hypertension (PH), a disease characterized by the remodeling of lung vasculature, which ultimately leads to right heart failure [61]. Frille et al. observed that the serotonin uptake is higher in the lungs of OSA patients with PH than those without [61]. Serotonin, apart from promoting vasoconstriction, promotes the proliferation of pulmonary arterial smooth muscle cells, thus contributing to PH [61].

To the best of our knowledge, this is the first study to comprehensibly explore the differences in the selected element of serotonergic signaling in the context of the mental health of OSA patients. It underlines the necessity for investigating the differences between OSA patients in order to be able to appropriately tailor therapy and address other, superficially unrelated health concerns, such as affective disorders, fatigue, or insomnia, supporting the conclusions presented in our previous work [21].

An important quality of the discussed undertaking is a relatively large number of participants (*n* = 76), which is even more crucial when considering rather difficult access to polysomnography. A combination of methodologies involving the assessment of both subjective parameters regarding well-being and objective evaluation of gene expression and protein synthesis gave a nuanced insight into the pathophysiology of OSA and the differences between potential phenotypes.

Despite promising results, there are several limitations to this study. First, the majority of patients suffered from severe OSA, which did not allow for further analysis between selected subsets of patients created based on the AHI.

Second, the majority of our study subjects were men. While male sex indeed is a risk factor for OSA, it is impossible to conclude whether similar changes would be seen in women, especially considering that they are disproportionally more affected by insomnia or depression [62,63].

Third, serotonin, SERT protein, and the expression of SERT mRNA were analyzed on the periphery, which limits the insight into changes occurring in the brain. Even though some studies attempted to show a link between depression and low circulating serotonin levels, there is no evidence supporting a direct causal relationship between the two [64]. Moreover, it is well established that peripheral serotonin does not bear any relationship to the level of serotonin within the central nervous system, as in a physiological state it cannot cross the blood–brain barrier [65]. Additionally, the molecular underpinnings of depression in the brain are more complex than a simple serotonin deficiency [66]. Nevertheless, considering the impact of this neurotransmitter on various aspects of functioning, including immunity, behavior, and appetite, further investigation of this parameter appears validated [67,68]. Nonetheless, when analyzing the results of studies such as this, it is necessary to note that peripheral and brain serotonin are two completely separate pools with distinctive roles.

In the case of SERT, even though its peripheral level is universally agreed to mirror changes occurring within the central nervous system, which validates projects such as the present one, there are controversies surrounding this subject [69]. Nevertheless, analysis of the SERT in blood might still reflect general tendencies, which renders it valuable. Moreover, as in the case of serotonin, disturbances in peripheral protein synthesis allow suspecting that it might also be affected within the central nervous system, which lays the groundwork for future studies.

This study provides a comprehensive exploration of serotonergic signaling in the context of OSA, focusing on its interplay with mental health, sleep disturbances, and QoL. While no significant differences were observed between OSA patients and controls in overall serotonergic parameters, distinctions became evident after stratification based on questionnaire results regarding life quality, depression, and insomnia.

The most important practical takeaway from this study is the benefit of stratifying OSA patients into phenotypes based on the state of their mental health, as well as objective biochemical markers. Such a personalized approach could allow for more personalized treatment strategies. For instance, the use of psychiatric medications to manage comorbid affective disorders may impact OSA parameters through their effects on serotonergic signaling. Moreover, the identification of morning serotonin and SERT protein levels as potential predictors for AHI and arousal, respectively, suggests that they could serve as supplementary tools in diagnosing and monitoring OSA severity.

Future research on serotonergic signaling in OSA should focus on exploring the central nervous system’s serotonin pathways by combining advanced neuroimaging techniques, protein and gene expression analysis, and the patient’s subjective health assessment via questionnaires. These methods would provide a deeper understanding of regional brain changes in serotonergic activity and their relationship with sleep disturbances, depression, and circadian dysregulation observed in OSA patients. Longitudinal studies are also warranted to track changes in serotonin, SERT protein, and mRNA expression over time, especially in response to standard OSA treatments like continuous positive airway pressure (CPAP) therapy. Furthermore, future research should aim to investigate potential pharmacological interventions targeting serotonergic pathways, assessing their efficacy in improving both sleep parameters and mental health outcomes in OSA patients. Studies incorporating larger, more diverse populations, including a balanced representation of genders and varying OSA severity levels, are essential for identifying sex-specific differences and refining therapeutic approaches.

## Figures and Tables

**Table 1:** Comparison of the study groups.

	OSA	CG	*p*
Women, %	11.4%	33.3%	**0.025**
Age	53.0 (45.0–67.0)	46.3 ± 12.3	**0.001**
BMI (kg/m^2^)	35.5 ± 6.5	27.7 (24.5–30.5)	**<0.001**
Sleep Efficiency (%)	83.3 (76.9–87.8)	83.8 (71.9–88.8)	0.908
Sleep Onset Latency (min)	18.3 (8.5–29)	24.5 (10.8–35.5)	0.237
Sleep Maintenance Efficiency (%)	87.5 (84.9–91.6)	87.2 (81.9–96.1)	0.877
REM Latency (min)	120.5 ± 67.3	110 (80.5–158)	0.713
Arousal Index (events/h)	23.8 (16.5–34.9)	9.3 (5.1–12.3)	**<0.001**
AHI (events/h)	51.5 (46.6–72.2)	1.5 (1.0–2.8)	**<0.001**
ODI (events/h)	61.0 ± 23.8	2.0 (1.0–3.0)	**<0.001**
AIS score	9.5 ± 4.3	10.1 ± 3.9	0.493
ISI score	13.3 ± 5.2	13.7 ± 5.4	0.606
BDI score	10.0 (6.0–15.0)	10.0 (8.0–17.0)	0.301
SF 36 score	73.4 ± 32.0	74.1 ± 27.3	0.923
A. SERT [pg/mL]	88.3 (76.2–107.9)	93 (74–118.7)	0.571
B. SERT [pg/mL]	325.2 (87.1–613.8)	221.1 (88.9–532.4)	0.696
A/B SERT	0.2 (0.2–1)	0.5 (0.2–1)	0.689
A. Serotonin [ng/mL]	85 (43–236.1)	72.4 (51–120.9)	0.331
B. Serotonin [ng/mL]	76.2 (44.6–158.1)	68.3 (43.2–143.1)	0.708
A/B Serotonin	1.1 (0.6–2.3)	1 (0.6–1.4)	0.759
A. SERT mRNA	22.0 (12.7–51.2)	15.6 (7–32.1)	0.099
B. SERT mRNA	23.7 (11.9–38.8)	21.7 (8.5–30)	0.286
A/B SERT mRNA	1.1 (0.6–1.8)	0.9 (0.3–1.8)	0.506

Data were presented as median (IQR) or mean ± standard deviation, depending on the distribution (non-normal or normal, respectively). A/B SERT—ratio of evening to morning SERT protein levels; A/B Serotonin—ratio of evening to morning serotonin levels; A. Serotonin [ng/mL]—evening serotonin concentration in nanograms per milliliter; A. SERT [pg/mL]—evening SERT protein concentration in picograms per milliliter; AHI—apnea/hypopnea index, events per hour; AIS—Athens Insomnia Scale score; B. Serotonin [ng/mL]—morning serotonin concentration in nanograms per milliliter; B. SERT [pg/mL]—morning SERT protein concentration in picograms per milliliter; BDI—Beck Depression Inventory score; BMI—body mass index, kg/m^2^; CG—control group; ISI—Insomnia Severity Index score; ODI—oxygen desaturation index, events per hour; OSA—obstructive sleep apnea; REM—rapid eye movement; SF-36—Short-Form 36 Health Survey score. Bolded text indicates statistical significance.

**Table 2:** Analysis of differences in SERT and serotonin between groups based on questionnaire scores.

		*n*	A. SERT [pg/mL]	B. SERT [pg/mL]	A/B SERT	A. Serotonin [ng/mL]	B. Serotonin [ng/mL]	A/B Serotonin	*n*	A.SERT mRNA	*n*	B. SERT mRNA	*n*	A/B SERT mRNA
OSA	ISI > 14	14	88.3 (75.6–91.6)	357.4 (83.4–609.2)	0.2 (0.2–1.0)	103.5 (70.9–229.5)	56.3 (44.9–130.8)	1.2 (0.7–4.5)	11	24.0 (17.0–64.2)	11	24.3 (13.4–38.8)	9	0.9 (0.6–1.3)
	ISI ≤ 14	21	95.2 (77.0–120.8)	331.2 (90.8–618.5)	0.2 (0.2–1)	87.8 (47.8–242.7)	114.1 (46.9–213.2)	1.1 (0.5–1.7)	21	21.4 (10.2–47.8)	20	19.1 (9.8–37.7)	20	1.2 (0.7–1.9)
*p*			0.434	0.987	0.606	0.583	0.454	0.293		0.481		0.317		0.532
CG	ISI > 14	15	106.6 (72.4–130.2)	175.6 (84.0–648.8)	0.8 (0.2–1.3)	0.8 (0.2–1.3)	66.7 (40–134.7)	1 (0.5–1.6)	12	7.2 (2.8–19.4)	15	20.1 (6.1–29.0)	12	0.5 (0.2–1.2)
	ISI ≤ 14	24	90.3 (72.9–105.5)	273.1 (92.2–501.3)	0.3 (0.2–0.9)	74.7 (52.4–128.2)	72.4 (43.5–162)	1 (0.6–1.4)	22	24.2 (11.8–33.6)	24	22.9 (13.2–31.3)	22	1.0 (0.7–3.0)
*p*			0.155	0.743	0.809	0.432	0.368	0.721		0.063		0.658		**0.037**
OSA	BDI > 13	11	91.6 (87.7–118.5)	525 (72.3–609.2)	0.2 (0.2–1.3)	125.0 (50.9–213)	114.1 (46.9–149.1)	0.8 (0.7–2.4)	9	18.0 (15.5–24)	11	24.3 (13.3–38.8)	9	0.9 (0.5–1.3)
	BDI ≤ 13	24	82.2 (75.3–101.0)	245.2 (87.1–626.0)	0.3 (0.2–1)	85.0 (42.5–272.6)	76.2 (44.6–231.2)	1.1 (0.5–2.3)	23	32.0 (10.2–64.2)	20	22.8 (11.2–37.7)	20	1.2 (0.7–1.9)
*p*			0.186	0.612	0.662	0.766	0.766	0.793		0.341		0.611		0.317
CG	BDI >13	15	81.1 (66.6–97.8)	467.7 (117.9–648.8)	0.2 (0.1–0.8)	75.8 (59.9–591.8)	69.8 (46.4–178)	1.3 (0.4–8.9)	14	10.7 (2.7–33.6)	15	27.2 (5.4–37.6)	14	0.5 (0.2–1.8)
	BDI ≤ 13	24	101.4 (84.3–126.8)	149.4 (80.6–501.9)	0.8 (0.2–1.2)	68.7 (40.5–79)	64.7 (41.1–132.7)	1 (0.7–1.2)	20	17.7 (11.6–31.7)	24	21.7 (9.8–28)	20	1.0 (0.7–2.1)
*p*			**0.044**	0.091	**0.005**	0.081	0.484	0.223		0.377		0.679		0.192
OSA	SF 36 < 64	10	82 (66.9–109.5)	141.9 (83.4–331.2)	0.5 (0.2–1)	42.3 (20.5–94)	43.9 (34.7–70.5)	1 (0.5–1.2)	9	38.3 (10.2–64.2)	8	19.1 (10.3–33.1)	8	1.2 (0.8–1.7)
	SF36 ≥ 64	22	93.4 (77–116.5)	502 (78.5–650.5)	0.2 (0.2–1)	124.1 (70.1–229.5)	124 (52.7–196.5)	1.1 (0.6–2.1)	20	20.6 (11.6–36.6)	20	20.0 (11.9–36.4)	18	1.2 (0.6–2)
*p*			0.269	0.176	0.163	**0.028**	**0.043**	0.675		0.594		0.709		0.495
CG	SF 36 < 64	12	92.8 ± 24.3	206.6 (83.1–497.1)	0.5 (0.2–1)	72.5 (41.8–98.5)	53.7 (40.3–141)	1 (0.7–1.3)	10	14.2 (7.8–32.1)	12	17.3 (10.2–28.1)	10	0.9 (0.3–4.2)
	SF 36 ≥ 64	22	97.4 ± 21.7	174.4 (93.8–542.1)	0.8 (0.2–1.2)	66.9 (56.6–135.5)	68.2 (43–103)	1.2 (0.6–1.6)	19	13.8 (2.9–27.6)	22	21.5 (6.5–37.6)	19	0.7 (0.2–1.6)
*p*			0.488	0.79	0.986	0.736	0.557	0.606		0.604		0.817		0.456

Data are presented as median (IQR) or mean ± standard deviation, depending on the distribution (non-normal or normal, respectively). Abbreviations: A/B SERT—ratio of evening to morning SERT protein levels; A/B Serotonin—ratio of evening to morning serotonin levels; A. Serotonin [ng/mL]—evening serotonin concentration in nanograms per milliliter; A. SERT [pg/mL]—evening SERT protein concentration in picograms per milliliter; B. Serotonin [ng/mL]—morning serotonin concentration in nanograms per milliliter; B. SERT [pg/mL]—morning SERT protein concentration in picograms per milliliter; BDI—Beck Depression Inventory score; CG—control group; ISI—Insomnia Severity Index score; OSA—obstructive sleep apnea; SF-36—Short-Form 36 Health Survey score. Bolded text indicates statistical significance.

**Table 3:** Association between selected OSA parameters, SERT, and serotonin.

	Correlation		Regression	
AHI				
B. SERT	R = 0.422	*p* = 0.010	b = 0.402	*p* = 0.030
A/B SERT	R = −0.339	*p* = 0.043		
	R^2^ = 0.131, F = 5.225, *p* = 0.030			
AHI nREM				
A. SERT	R = 0.396	*p* = 0.020		
B. SERT	R = 0.467	*p* = 0.005	b = 0.406	*p* = 0.032
B. Serotonin	R = 0.452	*p* = 0.007		
	R^2^ = 0.133, F = 5.131, *p* = 0.032			
Arousal index				
B. Serotonin	R = 0.435	*p* = 0.010	b = 0.578	*p* = 0.001
B. SERT	R = 0.353	*p* = 0.040		
	R^2^ = 0.308, F =13.039, *p* < 0.001			

Abbreviations: A/B SERT—ratio of evening to morning SERT protein levels; A—B SERT—difference between evening and morning SERT protein levels; A. Serotonin—evening serotonin concentration; A. SERT—evening SERT protein concentration; B. Serotonin—morning serotonin concentration; B. SERT—morning SERT protein concentration; AHI—apnea/hypopnea index; AHI nREM—apnea/hypopnea index during non-REM sleep; ODI—oxygen desaturation index; R—Pearson correlation coefficient; R^2^—coefficient of determination in regression analysis; b—regression coefficient; *p*—*p*-value indicating statistical significance.

## Data Availability

Data available on request from authors due to privacy concerns.

## Supplementary Materials

The following supporting information can be downloaded at: https://www.mdpi.com/article/10.3390/jcm14020445/s1, Table S1: Correlations between selected study parameters in the OSA group.
