# Supplementary material for: The Influence of Serotonergic Signaling on Quality of Life, Depression, Insomnia, and Hypoxia in Obstructive Sleep Apnea Patients: Cross-Sectional Study
Source: J Clin Med. 2025 Jan 12;14(2):445. doi: 10.3390/jcm14020445 (PMC11766041; doi:10.3390/jcm14020445)
Supplement: Supplementary file 1 [file jcm-14-00445-s001.zip › jcm-3403677-supplementary.pdf]

Table S1. Correlations between selected study parameters in the OSA group.

	A. SERT [pg/mL]			B. SERT [pg/mL]			A/B SERT			A. Serotonin [ng/mL]			B. Serotonin [ng/mL]			A/B Serotonin			A.SERT mRNA			B. SERT mRNA			A/B SERT mRNA		
	n	R	*p*	*n*	R	*p*	*n*	R	*p*	*n*	R	*p*	*n*	R	*p*	*n*	R	*p*	*n*	R	*p*	*n*	R	*p*	*n*	R	*p*
Age	35	−0.288	0.094	35	0.043	0.805	35	−0.086	0.622	35	0.231	0.182	35	0.082	0.639	35	0.266	0.122	31	−0.045	0.809	30	−0.062	0.746	28	0.023	0.908
BMI	**35**	**0.387**	**0.021**	35	0.296	0.084	35	−0.159	0.363	35	−0.027	0.879	**35**	**0.439**	**0.008**	**35**	**−0.370**	**0.029**	31	−0.123	0.510	30	−0.099	0.601	28	0.041	0.836
Sleep Efficiency (%)	20	−0.023	0.925	20	0.012	0.960	20	0.003	0.990	20	−0.027	0.910	20	−0.316	0.175	20	0.099	0.677	**19**	**0.472**	**0.041**	**16**	**0.791**	**<0.001**	16	−0.332	0.208
Sleep onset latency (min)	34	0.045	0.799	34	−0.147	0.408	34	0.174	0.326	34	0.174	0.324	34	0.033	0.851	34	0.238	0.175	32	−0.045	0.805	30	−0.074	0.698	29	0.102	0.598
Sleep Maintenance Efficiency (%)	20	−0.235	0.319	20	−0.170	0.474	20	0.129	0.587	20	0.083	0.729	20	−0.436	0.055	20	0.264	0.261	19	0.307	0.201	**16**	**0.686**	**0.003**	16	−0.358	0.174
REM latency (min)	34	−0.041	0.819	34	0.044	0.805	34	−0.011	0.951	34	0.073	0.682	34	−0.118	0.506	34	0.140	0.430	32	0.045	0.805	30	0.248	0.186	29	−0.169	0.381
REM% TST	34	0.011	0.951	34	0.058	0.745	34	−0.052	0.769	34	0.035	0.844	34	0.239	0.174	34	−0.066	0.709	32	0.157	0.390	30	0.087	0.647	29	0.083	0.669
nREM% TST	35	−0.015	0.931	35	−0.084	0.633	35	0.075	0.671	35	−0.101	0.563	35	−0.302	0.078	35	0.067	0.704	32	−0.157	0.390	30	−0.087	0.647	29	−0.083	0.669
Arousal index	34	0.162	0.359	**34**	**0.353**	**0.040**	34	−0.228	0.194	34	0.337	0.051	**34**	**0.435**	**0.010**	34	−0.049	0.782	32	−0.186	0.307	30	0.048	0.801	29	−0.134	0.490
AHI REM	33	0.142	0.429	33	0.241	0.177	33	−0.244	0.171	33	0.139	0.440	33	0.236	0.186	33	−0.122	0.498	32	−0.063	0.733	30	0.081	0.671	29	0.033	0.863
AHI nREM	**34**	**0.396**	**0.020**	**34**	**0.467**	**0.005**	**34**	**−0.355**	**0.040**	34	0.186	0.292	**34**	**0.452**	**0.007**	34	−0.181	0.306	**31**	**−0.375**	**0.038**	29	0.031	0.875	28	−0.315	0.102
AHI	36	0.314	0.063	**36**	**0.422**	**0.010**	**36**	**−0.339**	**0.043**	36	0.231	0.175	36	0.310	0.066	36	−0.075	0.663	32	−0.308	0.086	31	0.059	0.751	29	−0.262	0.170
Total number of desaturations	**22**	**0.552**	**0.008**	22	−0.025	0.911	22	0.177	0.431	22	−0.202	0.368	22	0.031	0.891	22	−0.250	0.262	19	−0.014	0.955	17	0.029	0.911	16	−0.015	0.957
Desaturation Index	**36**	**0.426**	**0.010**	**36**	**0.351**	**0.036**	36	−0.256	0.131	36	0.131	0.445	36	0.286	0.091	36	−0.150	0.382	32	−0.262	0.147	31	−0.021	0.913	29	−0.162	0.402
ISI score	35	−0.054	0.756	35	0.092	0.600	35	−0.154	0.378	35	0.160	0.357	35	−0.163	0.351	35	0.229	0.186	32	−0.023	0.899	31	0.111	0.551	29	−0.104	0.593
BDI score	35	0.076	0.663	35	0.190	0.275	35	−0.243	0.160	35	0.239	0.167	35	0.181	0.299	35	0.061	0.727	32	−0.177	0.333	31	0.098	0.600	29	−0.196	0.308
SF 36 score	32	0.012	0.946	32	0.303	0.091	**32**	**−0.368**	**0.038**	**32**	**0.573**	**0.001**	32	0.302	0.093	32	0.315	0.079	29	−0.025	0.898	28	0.155	0.431	26	−0.115	0.577

Abbreviations: A/B SERT—ratio of evening to morning SERT protein levels; A—B SERT—difference between evening and morning SERT protein levels; A. Serotonin—evening serotonin concentration in nanograms per milliliter; A. SERT—evening SERT protein concentration in picograms per milliliter; B. Serotonin—morning serotonin concentration in nanograms per milliliter; B. SERT—morning SERT protein concentration in picograms per milliliter; AHI—apnea/hypopnea index; AHI REM—apnea/hypopnea index during REM sleep; AHI nREM—apnea/hypopnea index during non-REM sleep; BMI—body mass index; BDI—Beck Depression Inventory score; REM latency—time to the first REM sleep period; REM% TST—percentage of total sleep time in REM sleep; nREM% TST—percentage of total sleep time in non-REM sleep; ISI score—Insomnia Severity Index score; SF-36—Short-Form 36 Health Survey score; R—Pearson correlation coefficient; *p*—*p*-value indicating statistical significance. Bolded text indicates statistical significa
